# Neural signatures of visuo-motor integration during human-robot interactions

**DOI:** 10.3389/fnbot.2022.1034615

**Published:** 2023-01-26

**Authors:** Silvia Marchesotti, Fosco Bernasconi, Giulio Rognini, Marzia De Lucia, Hannes Bleuler, Olaf Blanke

**Affiliations:** ^1^Laboratory of Cognitive Neuroscience, Center for Neuroprosthetics and Brain Mind Institute, Ecole Polytechnique Fédérale de Lausanne, Geneva, Switzerland; ^2^Laboratory of Robotic Systems, Ecole Polytechnique Fédérale de Lausanne, Lausanne, Switzerland; ^3^Laboratoire de Recherche en Neuroimagerie, University Hospital (CHUV) and University of Lausanne (UNIL), Lausanne, Switzerland; ^4^Department of Clinical Neurosciences, Faculty of Medicine, University Hospital, Geneva, Switzerland

**Keywords:** robotics, electroencephalography, virtual reality, visuo-motor integration, sense of agency, bimanual movements, somatosensory evoked potentials, source imaging

## Abstract

Visuo-motor integration shapes our daily experience and underpins the sense of feeling in control over our actions. The last decade has seen a surge in robotically and virtually mediated interactions, whereby bodily actions ultimately result in an artificial movement. But despite the growing number of applications, the neurophysiological correlates of visuo-motor processing during human-machine interactions under dynamic conditions remain scarce. Here we address this issue by employing a bimanual robotic interface able to track voluntary hands movement, rendered in real-time into the motion of two virtual hands. We experimentally manipulated the visual feedback in the virtual reality with spatial and temporal conflicts and investigated their impact on (1) visuo-motor integration and (2) the subjective experience of being the author of one's action (i.e., sense of agency). Using somatosensory evoked responses measured with electroencephalography, we investigated neural differences occurring when the integration between motor commands and visual feedback is disrupted. Our results show that the right posterior parietal cortex encodes for differences between congruent and spatially-incongruent interactions. The experimental manipulations also induced a decrease in the sense of agency over the robotically-mediated actions. These findings offer solid neurophysiological grounds that can be used in the future to monitor integration mechanisms during movements and ultimately enhance subjective experience during human-machine interactions.

## Introduction

Over the past years, continuous advances in the fields of robotics and virtual reality (VR) have made human-machine interactions increasingly widespread in our society. Rehabilitation robotics (Klamroth-Marganska et al., 2014), upper-limb neuroprostheses (Borton et al., 2013; Shokur et al., 2021), surgical robotic interfaces (Hussain et al., 2014; D'Ettorre et al., 2021), and manipulators for industrial applications (Ajoudani et al., 2018) represent concrete examples of how robotic technology can be applied to enhance human manipulation skills and to recover sensorimotor functions in patients affected by motor impairments. This rapid expansion has led to the emergence of a new interdisciplinary field, bringing together cognitive neuroscience, VR and robotics, providing a new approach to study cognitive functions (Rognini and Blanke, 2016; Beckerle et al., 2019; Wilf et al., 2021) and to investigate abnormal mental states (Blanke et al., 2014; Salomon et al., 2020; Bernasconi et al., 2021). However, despite the latest technological progresses, current knowledge of the perceptual, sensorimotor and neural mechanisms involved in these sophisticated human-robot interactions remains limited. This might be due to the fact that most of the efforts have so far been dedicated to the “machine” side, to improve control and usability, but less to the understanding of the brain mechanisms involved in robotically-mediated interactions. To fill this gap, a dedicated line of work has exploited behavioral measures of multisensory integration of bodily cues to successfully characterize robotically-mediated interactions in a perceptually-grounded fashion (Ionta et al., 2011; Blanke, 2012; Sengül et al., 2012, 2013, 2018; Rognini et al., 2013; Salomon et al., 2013; Pfeiffer et al., 2014; Akselrod et al., 2021). Some of these approaches consists in measuring how information related to a robot is processed as similar to information related to the user's body (therefore how the two sources are integrated), as an implicit measure of easiness and effectiveness of human-robot interaction (Sengül et al., 2012, 2013, 2018; Rognini et al., 2013). A way to experimentally address this aspect is the use of a well-known paradigm in cognitive science, the cross modal congruency task, which has been extensively employed to investigate visuo-tactile spatial integration (Maravita and Iriki, 2004). In the context of human-robot interaction, this task allowed characterizing several aspects of human-robot interaction in terms of visuo-motor and visuo-tactile integration (Rognini et al., 2013; Romano et al., 2015) as well as visuo-proprioceptive integration (Sengül et al., 2013). Importantly, those studies tackled subjective aspects such as the sense of agency [i.e., the subjective experience of controlling one's own actions (Haggard, 2017)] and the feeling of a presence (Blanke et al., 2014). Despite these insights, the neural correlates of robotic interactions under dynamic conditions (i.e., during active body movements) remain largely unknown.

Here we used a robotic VR set-up (Rognini et al., 2013) to characterize the neural correlates of visuo-motor integration and the link with the sense of agency during self-generated and robotically-mediated interactions. We used a bimanual haptic interface, originally developed as a prototype device of a robotic surgery manipulator (DaVinci System; Sengül et al., 2012, 2013; Rognini et al., 2013) to track hands movement trajectory while a realistic visual feedback was provided through VR. To interfere with visuo-motor integration mechanisms, we manipulated the visual feedback in selected trials by introducing spatial (change in the movement direction from horizontal to vertical) and temporal (600 ms delay) mismatches between the executed movement and the one observed by the participant in the VR environment. In addition to multisensory integration, these experimental conflicts allow investigating modulations in the subjective sense of agency over these self-generated actions. The neural correlates of visuo-motor integration were identified by recording somatosensory evoked potential (SEP) to stimulation of the median nerve at the wrist while participants performed voluntary bimanual hand movements through the interface. The measurement of SEPs is a well-established method to probe neural activity in the context of multisensory integration, having being employed to study interactions between somatosensory cues and other sensory modalities such as auditory (Foxe et al., 2000; Kisley and Cornwell, 2006; Touge et al., 2008), visual (Schürmann et al., 2002; Sebastianelli et al., 2022), and visuo-motor (Bernier et al., 2009). In our study, the choice of a somatosensory stimulation was motivated by a large body of literature showing that somatosensory pathways are prominently involved in processing sensory-motor stimuli (Huttunen et al., 1996; Forss and Jousmaki, 1998; Avikainen et al., 2002) and modulated by self-generated movements (Blakemore et al., 2000; Wasaka and Kakigi, 2012; Macerollo et al., 2018). Thus, changes in SEPs induced by experimentally manipulating the visual feedback would inform us regarding which brain regions are involved in the integration of visual and motor cues. We expect the involvement of multimodal brain regions such as parietal and prefrontal association areas, according to longstanding evidence in this context (Quintana and Fuster, 1993; Goodale, 1998; Fogassi and Luppino, 2005; Iacoboni, 2006; Kanayama et al., 2012; Limanowski and Blankenburg, 2016).

In addition, SEP modulations have also been linked to changes in the subjective experience related to one's own body relying on multisensory integration mechanisms (Dieguez et al., 2009; Aspell et al., 2012; Heydrich et al., 2018; Palluel et al., 2020). We predict a decrease in the reported sense of agency when the visual feedback is experimentally manipulated as compared to when no conflict is induced, in accordance with a previous study employing the same robotic interface (Rognini et al., 2013). Crucially, activity in associative areas have been also widely associated with the sense of agency and other fundamental aspects of bodily self-consciousness (David et al., 2008; Blanke, 2012).

## Materials and methods

### Participants

A total of 13 individuals (three females and 10 males, mean age 23.4 years, SD ± 1.5, range 21–27) participated in this study but data from three of them was discarded due to poor signal quality or absence of somatosensory evoked components when averaged according to the stimulation onset. All participants had normal or corrected to normal vision, and had no history of neurological or psychiatric conditions. All participants gave written informed consent and were compensated for their participation. The study protocol was approved by the local ethics research committee—*Commission cantonale (VD) d'éthique de la recherche sur l'être humain*—and was performed in accordance with the ethical standards laid down in the declaration of Helsinki.

### Experimental procedure

In this study we combined robotics, VR and high-density electrical source imaging to study visuo-motor integration and the sense of agency during robotically-mediated interactions. Participants were instructed to perform continuous horizontal hands movements while interacting with a bimanual haptic interface during somatosensory evoked potentials (SEP) recording.

#### Robotic interface and virtual reality environment

**Figure 1:**
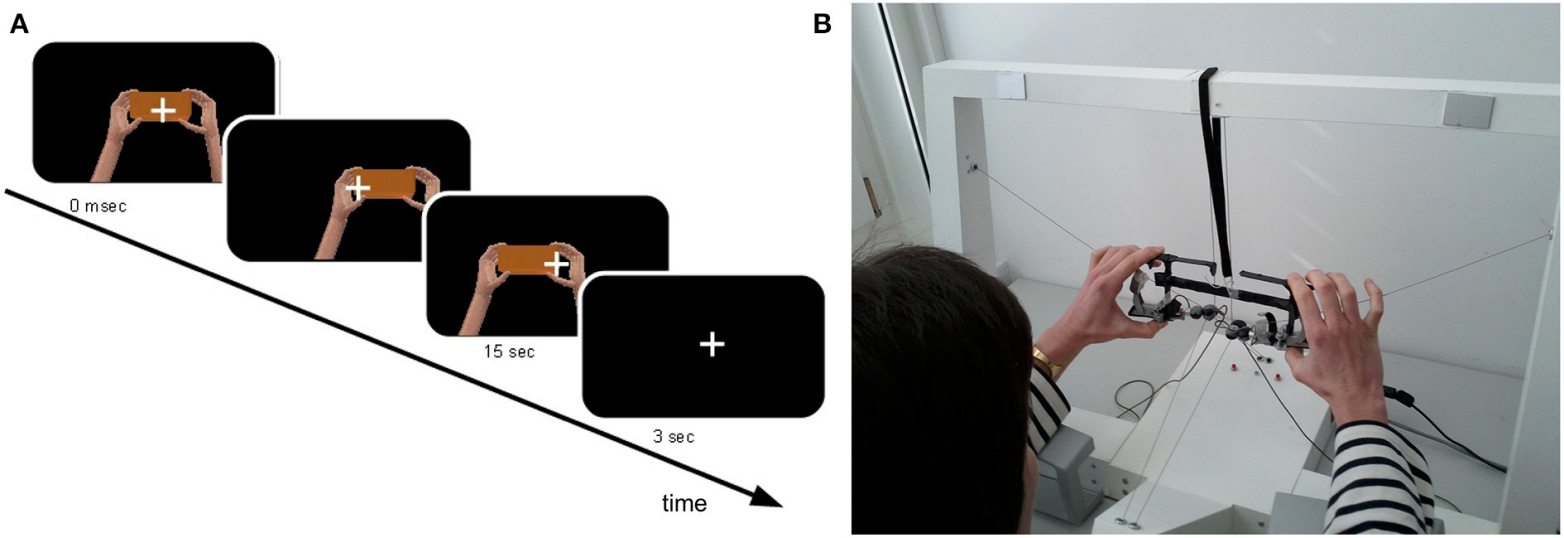
Experimental set-up and virtual reality environment. Participants sat at a table and hold the frame of the robotic device between the index and the thumb of each hand while performing continuous horizontal (left-right) hand movement for 15 s. **(A)** Participants' hands movements were translated in real time into the movement of two virtual hands, presented through a head-mounted display. To mimic the reality, the virtual hands were shown holding a block **(B)**.

As in a previous study conducted in our laboratory (Rognini et al., 2013), a bimanual haptic interface was exploited for tracking hands movements in real-time. This platform has been designed as a training device for users of the Da Vinci Surgical System (Intuitive Surgical, Sunnyvale, CA, USA), a robotic platform used to perform minimally invasive surgical procedures (Lanfranco et al., 2004). For each hand, this haptic interface allows seven degrees of freedom in motion and force feedback in three translations (Kenney et al., 2009; Lerner et al., 2010). Two grippers held by the participant are connected through cables to the motors, so that the force feedback and the movement tracking is performed through a cable-driven system. To maintain a fixed distance between the hands (20 cm) and between the thumbs and the index (8 cm) and give the impression of holding a cube, the two grippers were linked through a mechanical frame (24 × 8 cm), constructed with lightweight material (~100 g). During the experiment, participants sat at a table where the haptic interface was placed, holding the grippers with their hands and with the head supported by a chin rest to minimize head movement. The movements performed interacting with the haptic device were presented in real-time (except during the asynchronous condition, see below) in a virtual environment through a head-mounted display (HMD, eMagin Z800 3DVisor, ~40° angle view, 1.44-megapixel resolution, 50 cd/m^2^ brightness, 227 g). The compliance to the real-time condition, crucial for the investigation of multisensory integration, was already established in previous experiments employing the same robotics-VR set-up (Sengül et al., 2012, 2013; Rognini et al., 2013). To mimic at best the reality, the virtual scene showed two hands moving and holding a rectangular block (Figure 1A). To compensate for underestimation of the perceived distance in virtual reality occurring when HMDs are used, we used a scale factor (i.e., the ratio between the distance in virtual and physical world) of 1.5.

The virtual reality environment was developed with the open-source platform CHAI 3D (http://www.chai3d.org; Conti et al., 2003) and a set of C++ libraries. The original source code of CHAI3D can be found at: www.chai3d.org/download/releases, while the experimental paradigm main code can be found at the GitHub repository: github.com/sissymarche/SEP_VRMimic_Study.

#### EEG acquisition and median nerve stimulation

Continuous EEG was acquired at 2048 Hz through a 64-channel Biosemi ActiveTwo system (Biosemi, Amsterdam, Netherlands) referenced to the CMS-DRL ground. Electrodes were evenly spaced according to the 10–20 EEG system and included conventional midline sites Fz, Cz, Pz, Oz, and sites over the left and right hemispheres. This high-density EEG montage was chosen to perform source reconstruction and to allow us to investigate changes in the neural generators throughout the entire brain. Electrooculogram (EOG) was also recorded to control for eye movements.

The somatosensory stimulation was delivered to participants' median nerve at the right wrist using a bipolar transcutaneous stimulation (Grass S48, West Warwick, USA) able to produce square waves with a constant current output through an insulating unit (Grass SIU5 RF Transformer isolation unit, West Warwick, USA). The intensity of the stimulation was tuned separately for each participant: after having found the intensity required to achieve the abduction of the thumb, the current was reduced at the 80% of this motor threshold and the value was kept throughout the entire recording. The stimulation was delivered at a frequency of 2 Hz and with a duration of 0.2 ms to avoid electric artifacts on the recorded EEG traces. No participant reported pain or discomfort with this level of stimulation.

#### Procedure

Participants sat at a table and hold the frame of the robotic interface between the index and the thumb of each hand while performing continuous horizontal (left-right) hand movement (Figure 1B). They were asked to perform narrow trajectories, keeping the movements' velocity as constant as possible, avoiding sudden movements. Additionally, participants were instructed to keep their elbows lifted during the movement in order to avoid tactile cues coming from the contact with the table. A fixation cross was presented in the middle of the display to minimize eye movement. The median nerve stimulation, delivered at the right wrist, continued throughout the entire experiment without interruptions.

The VR environment (see Section Robotic interface and virtual reality environment) was presented right at the beginning of each trial through a head-mounted display: participants could start interacting with the robotic interface immediately after and continued performing horizontal movements till the end of the trial, for a total duration of 15 s. A 3 s inter-trial break allowed participants to rest; during this time the virtual environment was replaced by a black screen. Each session consisted in a total of four blocks, each of which encompass 16 trials (four repetitions/condition) arranged in a randomized order, and lasting ~5 min. A total of 480 somatosensory stimulations per condition were delivered to each participant.

We tested the effect of visuo-motor integration on SEP and the sense of agency by experimentally manipulating the visual feedback provided through VR with two types of visuo-motor conflicts. During selected trials, the visual feedback was perturbed in real-time by changing the actual movement direction from horizontal (left-right) to vertical (up-down, *congruency* factor) and by introducing a 600 ms delay (*synchrony* factor) between the actual and the seen movements. This temporal delay was chosen in accordance with a previous study employing the same robotic interface (Rognini et al., 2013), and due to the strong effect elicited when using this duration on the subjective experience (Tsakiris et al., 2006). Therefore, the experiment included four experimental conditions arranged in a 2 × 2 factorial design with *congruency* (congruent/incongruent) and *synchrony* (synchronous/asynchronous) as factors. Out of the four conditions, randomly intermixed across trials, only in one condition the visual feedback reflected entirely the actual movement (congruent and synchronous).

At the end of the session, the sense agency for the seen movement during each of the four experimental conditions was assessed by means of one question concerning the perceived feeling of control (“*I felt as I was responsible for the movement in the virtual reality*”*)* and one control question (“*I felt as if my hands were turning virtual*”). This question allows to control for task compliance (as verified by differences in the average ratings to the different questionnaire items) and it has been effectively used for the same purpose in several VR-based studies (e.g., Slater et al., 2008; Perez-Marcos et al., 2009; Sanchez-Vives et al., 2010), including one from our group that employed the same robotic platform to address the sense of agency and ownership over virtual hands (Rognini et al., 2013). In our study, it is particularly suitable as a control item for the sense of agency question since it addresses perceptual aspects that are static rather than dynamic, these latter required to investigate the sense of agency. Participants had to answer by rating their subjective experience on a scale from 0 (strongly disagree) to 10 (strongly agree).

### Data analysis

#### EEG preprocessing

EEG data preprocessing was conducted using the EEGlab (Delorme and Makeig, 2004) and FASTER (Nolan et al., 2010) toolboxes within the MATLAB (The MathWorks) environment. Data were first down-sampled to 512 Hz, band pass filtered (1–40 Hz) and re-referenced to the average value. For SEP calculation, the EEG epochs were time-locked to the electrical stimulation onset and covered a time window of 50 ms pre-stimulus and 300 ms post-stimulus, using the pre-stimulus interval for baseline correction. Epochs with amplitude exceeding a threshold value (±20 μV) were excluded. Bad channels were interpolated using a spherical interpolation. Artifactual epochs were removed *via* visual inspection, and residual artifacts removed using independent component analysis (ICA).

#### Topographic analysis of EEG data and source estimation

The analysis of the EEG signals consisted in a well-established multi-step procedure to investigate changes in the configuration of intracranial generators across conditions and was implemented mainly with the software Cartool (https://sites.google.com/site/cartoolcommunity/; Murray et al., 2008; Brunet et al., 2011). In-brief, first a topographical clustering algorithm is used to select periods of time, within each trial, in which brain activity is modulated by the experimental manipulation. Secondly, an inverse solution method is applied to evaluate differences in the neural generators of the signals recorded over the time periods found in the previous step across the experimental conditions. This procedure has been extensively described in previous works (Michel et al., 2004; Murray et al., 2008) and, being reference-independent and data-driven, offer analytical and interpretational advantages over canonical waveform analyses (Murray et al., 2008).

As a first step, the predominant topographies (maps) are identified by applying a Atomize and Agglomerate Hierarchical Clustering algorithm to the group-averaged data across all conditions. This approach consists in considering initially all possible clusters (one for each data point) and through subsequent iterations, reducing their number based on their contribution to the global explained variance (GEV). The optimal number of template maps is then found by applying a modified version of the Krzanowski–Lai criterion (Pascual-Marqui et al., 1995; Murray et al., 2008). Here we considered solutions with topographies accounting for at least 90% of the global dataset variance. The pattern of template maps found at the group-level is then statistically evaluated using single-subject data in a procedure referred to as “fitting.” This consists in computing the spatial correlation between the template maps and single-subject data at each time-point and each experimental condition. From this fitting procedure we extracted, for each condition and participant, the maps' presence (in milliseconds) and the associated global field power (GFP), two orthogonal features of the recorded electric field. GFP is a reference-independent measure, computationally equivalent to the standard deviation of all electrodes at a given time, thus its value is proportional to the strength of the electric field.

Map duration and GFP values were then submitted to a permutation-based repeated measures 2 × 2 ANOVA with *congruency* and *synchrony* as factors and 10,000 resampling. This non-parametric analysis was carried out using the “Permuco” R package, a statistical approach more appropriate than parametric ANOVA when the assumption of normality is violated or with moderate size datasets (Frossard and Renaud, 2021). This analysis allowed us to investigate potential map specificities for a given condition and consequently possible differences in the underlying intracranial generators.

In order to estimate periods of time along which an evoked activity could be reliably measured, we carried out a topographic consistency test (TCT; Koenig and Melie-García, 2010), able to identify time periods in which it is possible to observe a consistent relationship between the somatosensory stimulation and neural electric sources. This analysis is based on the argument that the GFP of the average response across participants depends on the consistency between the individual-subjects' topographies. That is, only if topographies are similar across participants, it is possible to obtain a GFP of the grand-average higher than what could be observed if topographies contained mainly noise. This is tested by simply shuffling the measured potentials across electrodes in each topographies, and compute the probability to obtain a GFP larger or equal to the empirical one (Michel et al., 2009). Of note, the TCT does not aim at investigating differences among experimental conditions, as the test is performed separately for each condition.

We then estimated the intracranial sources over the time periods in which we found a significant topographical modulation and presenting stable topography according to the TCT, using a distributed linear inverse solution and applying the local autoregressive average regularization approach (LAURA; Grave De Peralta Menendez et al., 2001, 2004). This method is used to address the non-uniqueness of the inverse problem and it is based on the biophysical principle that the potential recorded on the scalp decays as a function of the square distance to the source. Its spatial accuracy has been evaluated as superior by previous studies comparing different inverse solution techniques using real and simulated data (Grech et al., 2008; Carboni et al., 2022). In our study we used a solution space including 4,996 nodes, selected from a 2 × 2 × 2 mm grid equally distributed within the Montreal Neurological Institute's (MNI) average template brain. As assessed in previous studies, the localization accuracy is considered to be along the lines of the matrix grid size, here being equal to 2 mm (Grave De Peralta Menendez et al., 2004; Martuzzi et al., 2009). Before the estimation of the neural generators, the SEP data in the temporal domain was first averaged across the time window found in the previous topographic pattern analysis, in order to generate a single data-point for each participant and condition, thereby increasing the signal-to-noise ratio (Thelen et al., 2012). The result of the source estimation gave a current density value for each node of the solution space that can be statistically tested to investigate significant differences in the brain sources between conditions. A 2 × 2 repeated measures ANOVA with factors *congruency* and *synchrony* was performed at each node using the STEN toolbox, developed by Jean-François Knebel and Michael Notter (http://doi.org/10.5281/zenodo.1164038). Only nodes with *p*-values < 0.05 and clusters of at least 17 contiguous nodes were considered statistically significant, using the same spatial threshold determined in previous studies (Cappe et al., 2012; Thelen et al., 2012).

#### Analysis of behavioral data

All behavioral data (questionnaires and movement parameters) were analyzed using a permutation-based repeated measures 2 × 2 ANOVA with 10,000 resampling with *congruency* and *synchrony* as factors. Reports for the control question from two participants were missing, therefore, to control for the smaller sample size we performed the same analysis on a pool or participants taking into consideration ratings from participants whose EEG was excluded for the analysis (*n* = 11) and compare it with the one obtained including the final pool (*n* = 8).

Correlation analyses were performed between significant changes in neural generators and the corresponding differences in the reported sense of agency (Pearson correlation coefficient).

Raw movements recorded through the robotic interface were analyzed to investigate whether, despite the explicit instruction to keep the horizontal movement as consistent as possible throughout the entire duration of the experiment, our experimental manipulations of the visual feedback could have affected the executed movement. We considered two parameters: the velocity and the trajectory norm, computed as the Euclidean norm of each left-right path (as in Rognini et al., 2013). For each participant we then computed and analyzed the average values across experimental conditions.

## Results

### Electrical neuroimaging results

**Figure 2:**
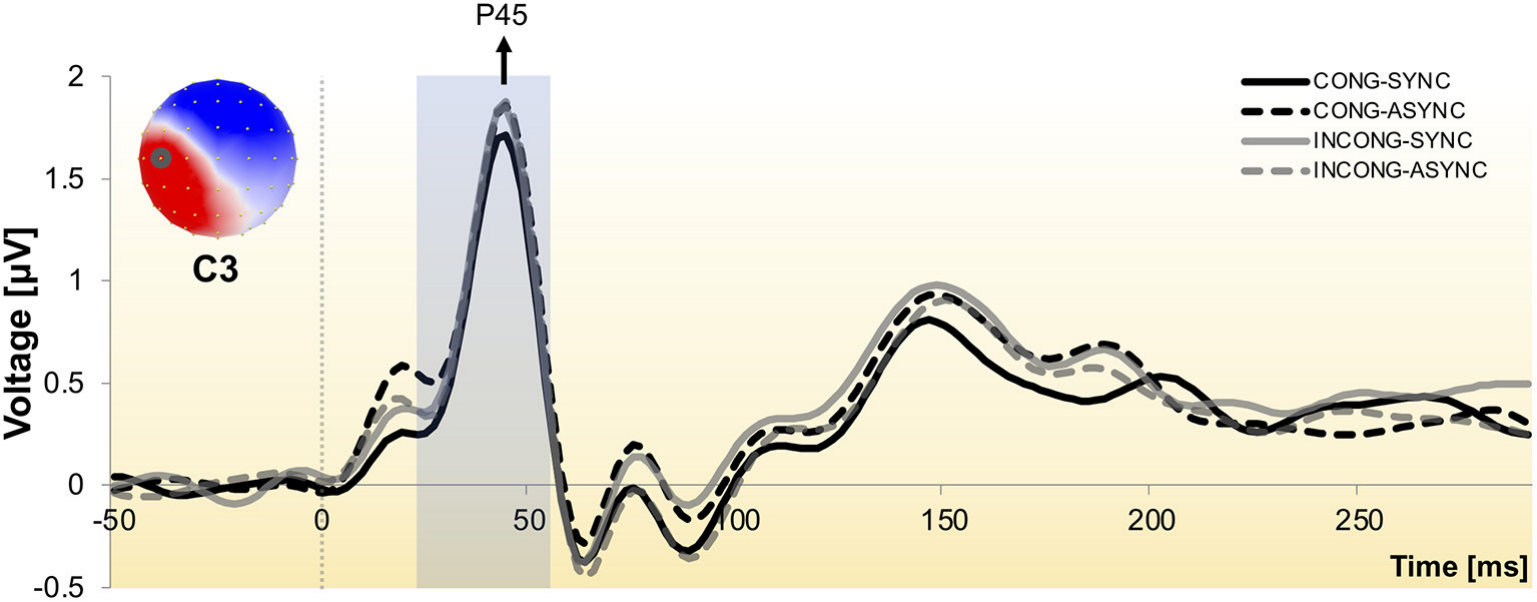
Somatosensory evoked potential to stimulation of the median nerve at the right wrist. Group-averaged SEP waveform across the four experimental conditions as recorded from one exemplar central electrode (C3), contralateral to the stimulation side. Responses exhibit the prototypical peaks, including the P45 and N60 components. The scalp topography is shown with the nasion upwards and left scalp leftwards and depicts the neural activity at the P45 peak, with electrode C3 encircled. The shaded time interval indicates the window of interest over which the neuroimaging analyses were performed.

The waveform analysis revealed the prototypical components evoked by median nerve stimulation at the wrist (e.g., P45, N60), with maximal amplitude displayed by electrodes placed over central and parietal regions, contralateral to the stimulation side (Figure 2).

**Figure 3:**
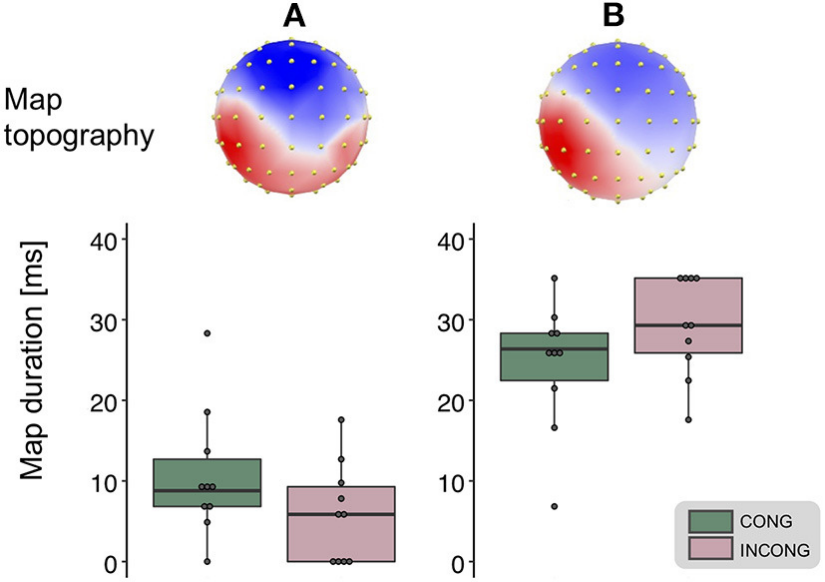
Topographic clustering and “fitting” procedure. The topographic pattern analyses identified periods of stable topography across the collective 300 ms post-electrical stimulation onset. For one of these time periods (21–56 ms), two maps were identified from the group-averaged SEPs. One map most prominently accounted for the congruent condition [map **(A)**, left], while the other for the incongruent one [map **(B)**, right]. The reliability of this result, observed at the group-average level, was assessed at the single-subject level using a spatial correlation fitting procedure. This analysis revealed a significantly different duration for the two maps in the congruent (green) as compared to the incongruent (pink) condition (main effect of the *congruency* factor, whiskers plots).

**Figure 4:**
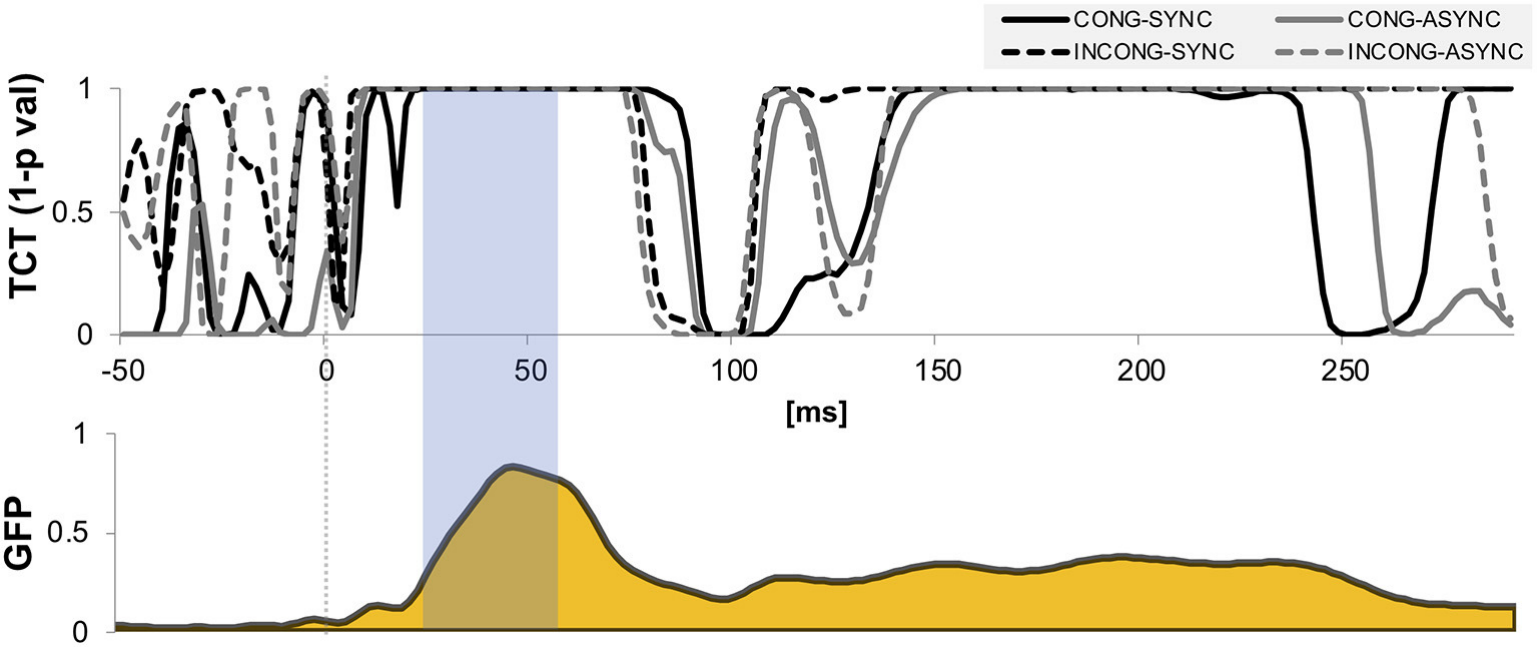
Topographic consistency and global field power. Results of the topographic consistency tests (TCT, upper plot) show intervals of time in which the null hypothesis of observed topographies explained entirely by noise is rejected (*p*-values < 0.05). For displaying purposes, the y-axis is expressed in expressed in terms of 1-*p* values. The TCT across the four conditions show periods of stable topographies during two time-windows, respectively 20–75 and 150–220 ms post stimulus onset. The first-time window covers the onset and peak of global field power (GFP, lower plot), and occurs around the P45 evoked potential. The time interval highlighted indicates the interval of interest over which the neuroimaging analyses were performed.

We performed hierarchical topographic clustering to identify periods of stable electric field topographies over the group-averaged data from all conditions. The topographic clustering accounted for 91% of the global explained variance and identified several periods of stable electric field topographies, one of which included the 21–56 ms post-stimulus interval. This time interval encompassed two topographic maps, both displaying a clear lateralization over the left central and parietal regions, contralateral to the stimulation side (Figure 3). Interestingly, one of the two topographies was selectively present in the *congruent* and *synchrony* condition (i.e., the one without any experimental manipulation, Figure 3A), showing a stronger activity over right parietal regions as compared to the other topography, which was predominant during the other three experimental conditions characterized by a visuo-motor mismatch (Figure 3B). The 21–56 ms interval overlapped with a window of topographic consistency across all four conditions as assessed through the TCT and included the peak of the GFP (Figure 4), in accordance with the inverse relationship between GFP and the significance of TCT and confirming evidence of evoked activity during this early time-period across the four conditions of interest (Murray et al., 2008). In the following we focus on the statistical analysis of the topographic representation of the EEG signal during the 21–56 ms post-stimulus period.

The reliability of the topographic pattern observed at the group-level in the previous step was tested at the single-subject level using a spatial correlation “fitting” procedure. Statistical tests over the map presence confirmed the predominance of the more posteriorly bilateral map (map A) when the virtual movement was spatially congruent with respect to the executed one: over the 21–56 ms interval we found a main effect of the factor *congruency* over the map duration [*F*_(1,9)_ = 11.42, *p* < 0.01, η^2^_p_ = 0.56, Figure 3]. A trend toward the same direction was present for the factor *synchrony*, but did not reach statistical significance [*F*_(1,9)_ = 3.52, *p* = 0.09, η^2^_p_ = 0.28]. There was no statistically significant interaction between the two factors [*F*_(1,9)_ = 1.19, *p* = 0.3, η^2^_p_ = 0.12]. We then tested the average GFP over the time window of interest and found no significant difference between the experimental conditions (all *p*-value > 0.05).

**Figure 5:**
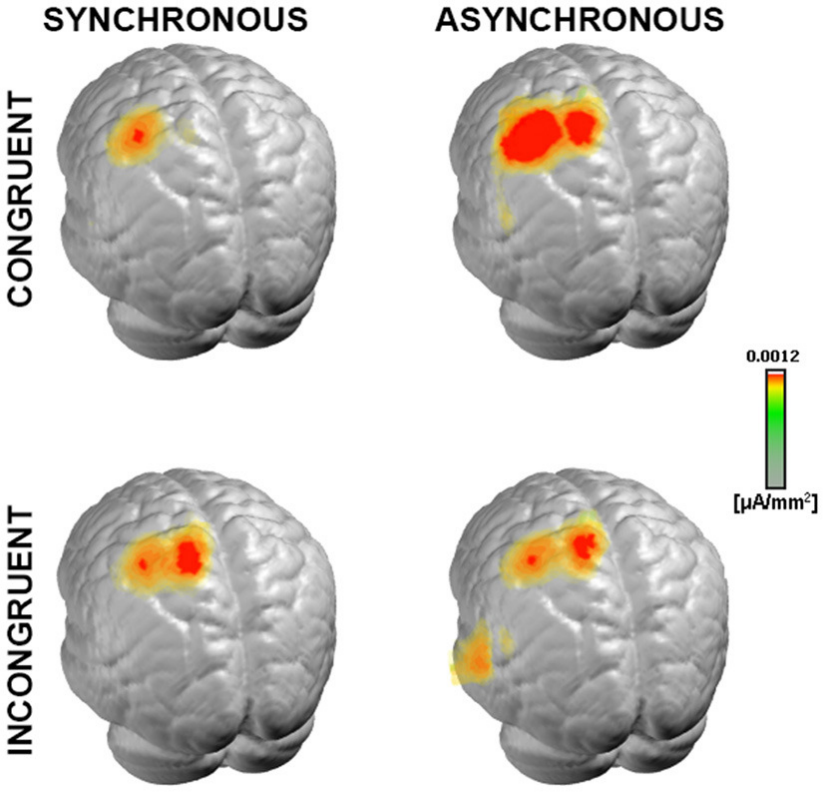
Neural generators of somatosensory evoked response. Group-averaged LAURA source density estimations over the 21–56 ms period post-stimulus onset for each of the four experimental conditions. Results are displayed on an average MNI brain. All conditions exhibited neural generators located within the left somatosensory area, contralateral to the stimulation side (right wrist).

Next, to test the hypothesis that such topographic differences reflect changes in the underlying neural generators, we computed and statistically compared the source estimations over the time interval of interest, across the four conditions. All experimental conditions presented neural generators located prominently over the left somatosensory cortex, contralateral to the stimulation side (Figure 5), as expected from the lateralized pattern observed at the scalp topography (Figures 2, 3).

**Figure 6:**
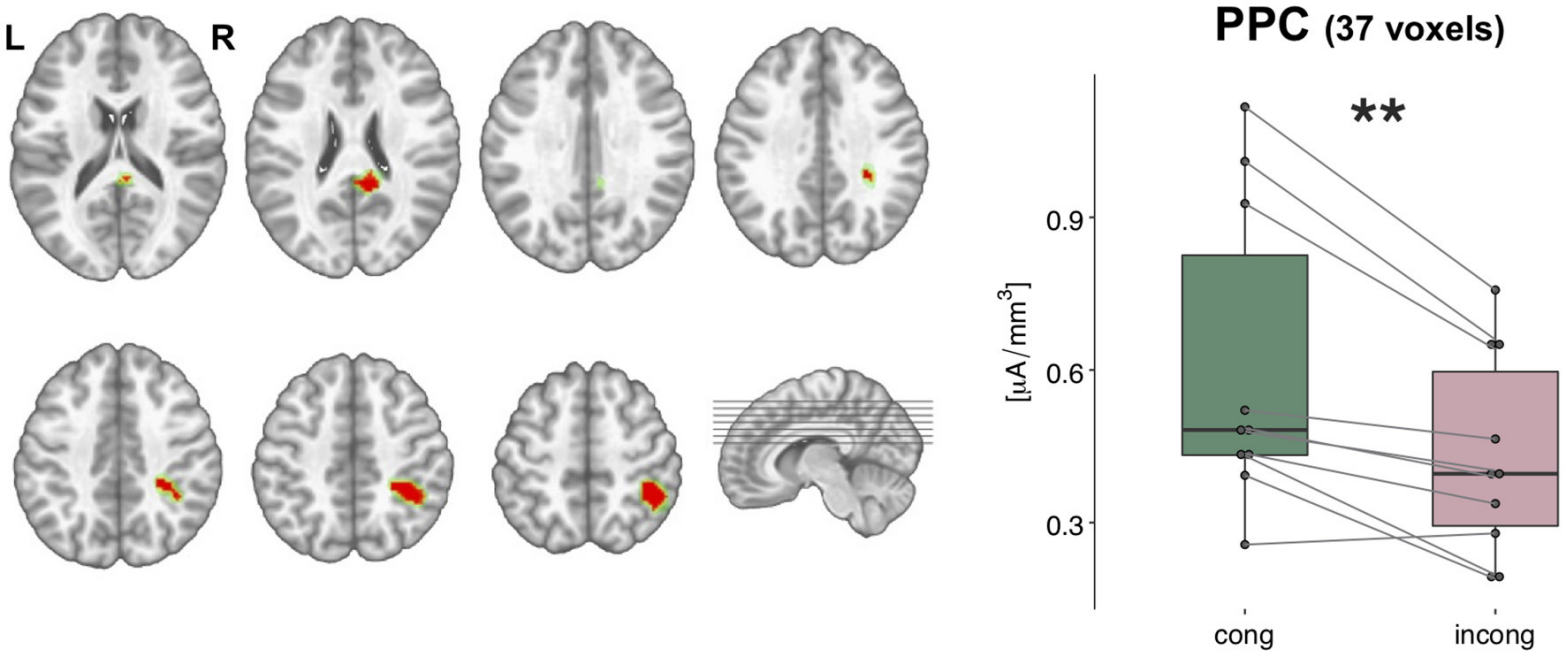
Statistical analyses of the source estimations. Group-averaged source estimations were calculated over the 21–56 ms post-stimulus interval for each experimental condition and submitted to a repeated measures 2 × 2 ANOVA performed in the brain space. Two clusters that exhibited a significant main effect of the factor *congruency* are shown in axial slices of the MNI template brain, in correspondence of the posterior parietal cortex (PPC, 37 nodes) and posterior cingulate cortex (PCC, 12 nodes). Only nodes meeting the *p*-values < 0.05 statistical threshold and the spatial criterion of at least 17 contiguous nodes were considered reliable. Whisker plots depict the current density values in the PPC cluster in the *congruent* (green) and *incongruent* (pink) conditions. Significance is denoted with ** for *p* < 0.01.

Scalar values of the current density of the sources from each participant and condition were statistically tested with a 2 × 2 ANOVA performed in the brain space. This analysis highlighted a cluster of 37 nodes in the posterior parietal cortex (PPC), where the estimated sources were stronger in the *congruent* condition as compared to the *incongruent* condition [*F*_(1,9)_ = 17.42, *p* < 0.01, η^2^_p_ = 0.66, Figure 6]. In addition, another cluster of 12 nodes over the posterior cingulate cortex (PCC) was significantly modulated for the main effect of *congruency*, but it will not be discussed any further since it did not survive the spatial threshold that we set.

### Behavioral data

#### Questionnaire analysis

**Figure 7:**
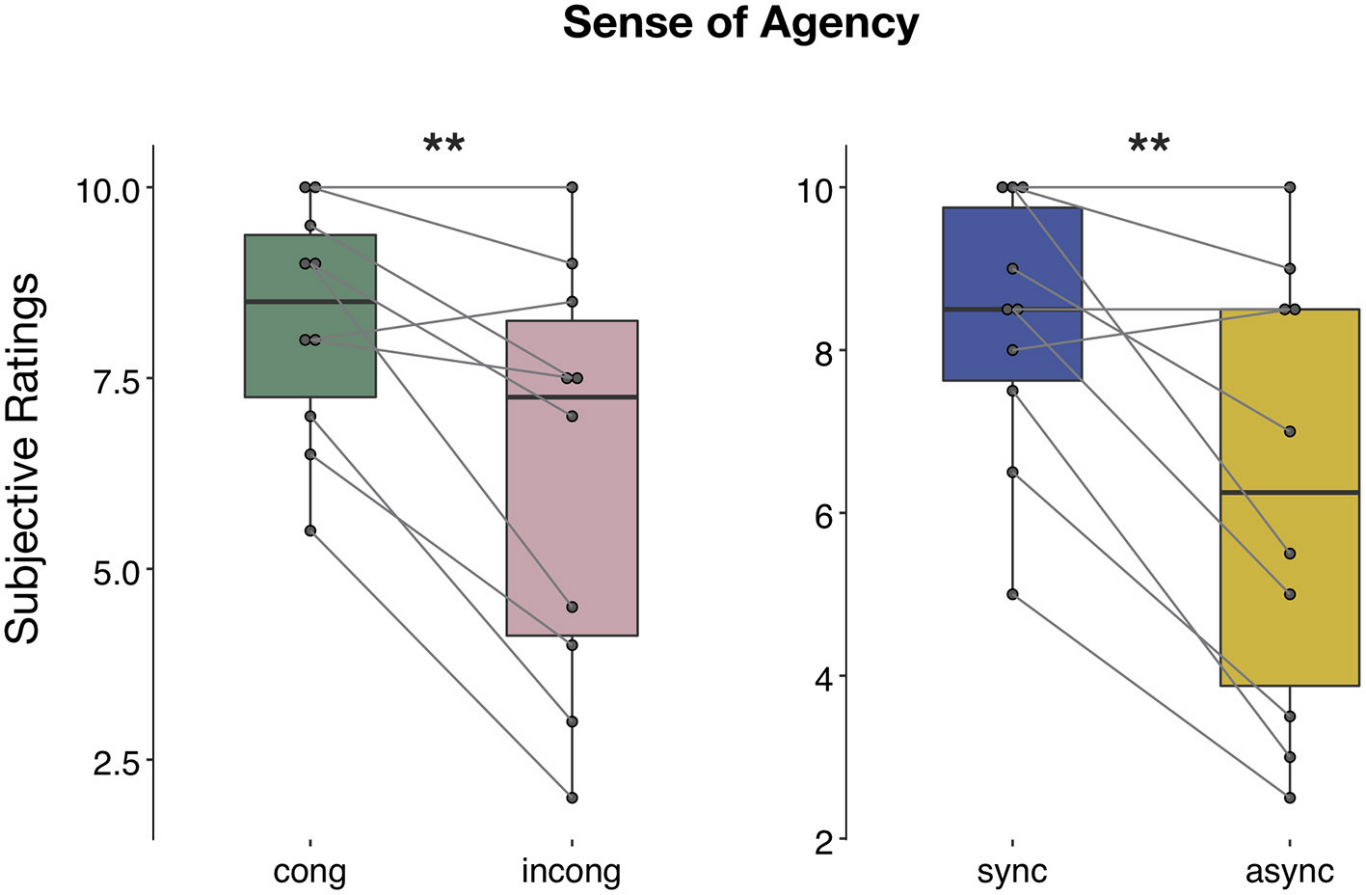
Sense of agency during human-robot interactions. Questionnaire results regarding the question on the sense of agency: the ANOVA revealed both a main effect of *congruency* and *synchrony*. Significance is denoted with ** for *p* < 0.01.

The ANOVA performed on the questionnaire scores on the sense of agency revealed a main effect of both *congruency* [*F*_(1,9)_ = 13.05, *p* < 0.01, η^2^_p_ = 0.59] and *synchrony* [*F*_(1,9)_ = 12.1, *p* < 0.01, η^2^_p_ = 0.57] factor, with participants reporting a stronger sense of agency during the *congruent* and *synchronous* conditions (Figure 7), but no significant interaction between the two factors [*F*_(1,9)_ = 1.12, *p* > 0.05, η^2^_p_ = 0.11]. Conversely, ratings for control question revealed no significant difference between the four conditions [main effect of *congruency*: *F*_(1,7)_ = 2.39, *p* > 0.05, η^2^_p_ = 0.26, main effect of *synchrony*: *F*_(1,7)_ = 0.02, *p* > 0.05, η^2^_p_ = 0.004, interaction *congruency* × *synchrony*: *F*_(1,7)_ = 1, *p* > 0.05, η^2^_p_ = 0.12; Supplementary Figure 1]. The absence of any statistical difference to the control questions was confirmed when considering a bigger sample size, including participants excluded from the EEG data analyses [main effect of *congruency*: *F*_(1,10)_ = 2.66, *p* > 0.05, η^2^_p_ = 0.21, main effect of *synchrony*: *F*_(1,10)_ = 0.003, *p* > 0.05, η^2^_p_ = 0.0003, interaction *congruency* × *synchrony*: *F*_(1,10)_ = 0.51, *p* > 0.05, η^2^_p_ = 0.05].

To investigate whether the neural changes observed in the PPC due to the effect of *congruency* factor might be related to the perceived SoA, we calculated the Pearson's *r*-value between the *congruent-incongruent* difference in current density and in the reported SoA and found no relationship between these two variables (*r* = 0.09, *p* > 0.05).

#### Movement trajectory

**Figure 8:**
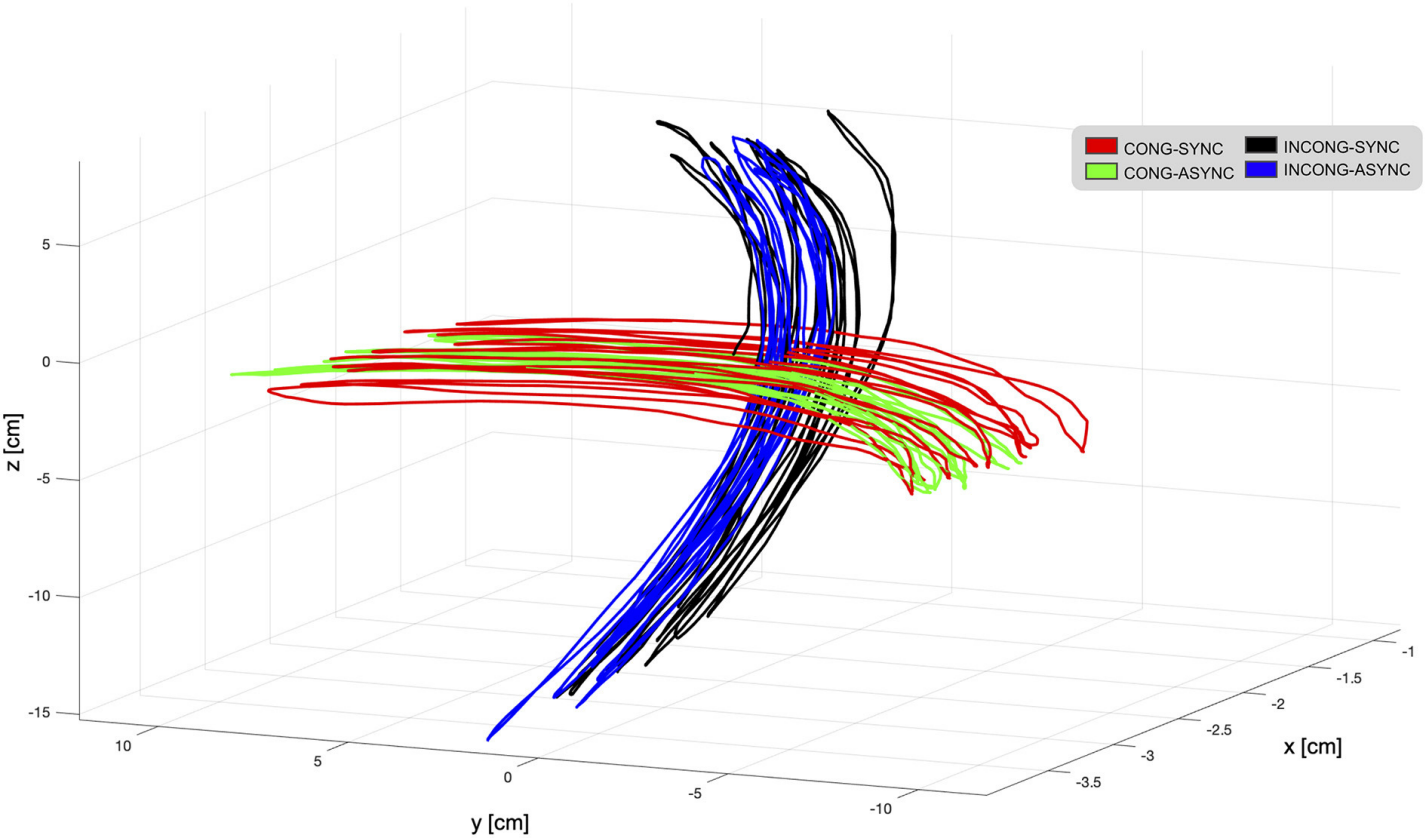
Movement trajectories as displayed in the virtual environment from one representative participant. During the task, participants were instructed to perform continuous horizontal left-right hand movements while interacting with the robotic interface. These movements were translated in real-time in the virtual reality environment, presented to the participant through a head-mounted display. To interfere with visuo-motor integration mechanisms, the visual feedback provided in the VR was either in accordance with the executed movement (congruent-synchronous condition, red trajectory) or experimentally manipulated. For this, a spatial mismatch translating the horizontal movement into a vertical one (incongruent conditions, black and blue trajectories) and a temporal delay (asynchronous conditions, green and blue trajectories) were employed in selected trials, resulting in a total of four experimental conditions. Trajectories are displayed for each condition during a single trial. Overall, the mean trajectory norm in the left-right direction was 10.6 cm, and the mean velocity 6.7 cm/s.

During the experiment, participants performed continuous movements with a mean trajectory norm of 10.6 cm (SE 0.6 cm) in the left-right direction, and with a mean velocity of 6.7 cm/s (SE 0.3 cm/s; Figure 8). Although there were discrepancies in the extent and velocity of the movements between participants, within-participant differences between conditions were minimal (below 1 cm for trajectory and below 1 cm/s for velocity in all participants except 2). This confirms that individual participants were able to perform the task in a consistent manner despite the presence of the experimental manipulation. The statistical analysis performed at the group level revealed a significant interaction of *congruency* and *synchrony* in the trajectory norm [*F*_(1,9)_ = 6.6; *p* < 0.05, η^2^_p_ = 0.42]. *Post-hoc* analyses revealed that, during the *incongruent-synchronous* condition, the trajectory was shorter than during the *incongruent-asynchronous* [*t*_(9)_ = 2.1, *p* = 0.05, *d* = 0.69] and the *congruent*-*asynchronous* [*t*_(9)_ = 1.85, *p* = 0.09, *d* = 0.58].

Statistical tests over average velocity revealed a main effect of *synchrony* [*F*_(1,9)_ = 34.8; *p* < 0.001, η^2^_p_ = 0.79] and a significant interaction between the two factors [*F*_(1,9)_ = 27.9; *p* < 0.001, η^2^_p_ = 0.75]. The movement was significantly slower during the delayed condition, irrespective of the presence of a spatial conflict [*incongruent-synchronous* > *congruent-asychronous*: *t*_(9)_ = 2.41, *p* < 0.05, *d* = 0.76, *incongruent-synchronous* > *incongruent-asychronous*: *t*_(9)_ = 2.86, *p* < 0.05, *d* = 0.9]. This finding is in line with the observation that, during the recordings, participants tended to slow down during the asynchronous trials when approaching the end of the right-left trajectory. Overall, manipulating the visual feedback resulted in a decreased velocity as compared to trials in which the feedback was in accordance with the executed movement [*congruent-synchronous* vs. *congruent-asynchronous*: *t*_(9)_ = 9.22, *p* < 0.001, *d* = 2.9; vs. *incongruent-synchronous*: *t*_(9)_ = 3, *p* < 0.05, *d* = 0.96; vs. *incongruent-asynchronous*: *t*_(9)_ = 4.55, *p* = 0.001, *d* = 1.44].

## Discussion

Our study aimed at identifying the neural correlates of visuo-motor integration during human-robotic interactions under dynamic conditions. To do so, we used a robotic interface to track bimanual movements and provided real-time visual feedback through VR (Rognini et al., 2013). This experimental set-up allowed us to introduce precise spatial and temporal mismatches between the executed and observed movement. To investigate how brain activity was modulated by these visuo-motor conflicts, we recorded somatosensory evoked potentials (SEPs) to stimulation of the median nerve at the right wrist. High-density electrical source imaging indicated that the spatial manipulation (*congruency* factor), but not the temporal one (*synchrony* factor), elicited a decrease in the neural activity in the right posterior parietal cortex (PPC). At the behavioral level, we observed that both spatial and temporal manipulations induced a significant decrease in the subjective experience of feeling in control over the movements of the virtual hands.

PPC has been traditionally considered as an associative area, integrating information from different sensory modalities (see for instance, Xing and Andersen, 2000). Several studies employing mismatches between the observed and felt movement through manipulation of the visual feedback consistently showed the involvement of this region in the detection of visuo-motor incongruences (Fink et al., 1999; Schnell et al., 2007; Wasaka and Kakigi, 2012). This region has also been proposed to be part of the fronto-parietal mirror neuron network, responsible for the correspondence between observed and executed movements (Rizzolatti and Craighero, 2004). Interestingly, we found that PPC is sensitive selectively to spatial rather than temporal conflicts. In line with this finding, PPC has been associated mainly to integration and processing of spatial information (Andersen and Zipser, 1988; Quintana and Fuster, 1993; Haggard, 2017), as well as sensory-spatial transformation processes (Torres et al., 2010), rather than temporal cues (Quintana and Fuster, 1993).

Moreover, the effect of the spatial manipulation was a stronger, and not reduced, activity in PPC when the observed movement was in accordance with the executed one, unlike previous studies reporting suppression of neural activity associated to self-generated actions or action attribution to the self (Fink et al., 1999; Farrer and Frith, 2002; Jeannerod, 2009). However, suppression mechanisms that appears at the behavioral level, such as the well-described inability to tickle oneself (Blakemore et al., 2000), and the physiological attenuation of SEP amplitude due to the gating effect during movement, have been shown to have different neurophysiological correlates (Palmer et al., 2016). Similar to our results, previous studies employing the same topographic analysis as the one we used also showed a stronger response as measured by evoked potentials when no experimental conflict was introduced as compared to a condition characterized by a visuo-tactile mismatch (Aspell et al., 2012). Authors have proposed that this phenomenon might be due to bottom-up multisensory integration mechanisms occurring when sensory cues are congruent, that would enhance the activity over modality specific regions such as the somatosensory cortex, as previously reported for visuo-tactile (Eimer and Driver, 2001; Taylor-Clarke et al., 2002) and sensorimotor integration through power increase in the gamma band (Aoki et al., 2001). In addition, enhanced activation over the intraparietal sulcus (IPL), a structure anatomically close to the cluster we found in our results, have been associated with integration processes of visual and spatial information in hand-centered coordinates (Makin et al., 2007).

Another important aspect of our findings is the lateralization to the right hemisphere, which cannot be ascribed to factors related to the task itself (the movement was bimanual) or differences in the executed movements (the analysis of the movement trajectory and velocity revealed no main effect of *congruency*). This effect follows a large body of literature supporting the prominent role of the right hemisphere in visuo-spatial processing. Previous studies have implicated the right PPC, rather than the left PPC, as a key site for visuo-spatial processing (Vallar, 1998) and monitoring of self- vs. externally- generated movements (Ogawa and Inui, 2007).

The analyses of the movements performed by participants highlighted differences between experimental conditions in the mean trajectory (interaction *synchrony* × *congruency*) and velocity (main effect of *synchrony*). These effects likely result from perceptual responses to mismatches in the visual feedback (e.g., slowing-down during the asynchronous condition to compensate for the temporal delay), and are not responsible for the changes observed at the neural level. It is true that active movements can modulate the waveform of SEPs, likely due to a top-down attenuation of the afferent proprioceptive information (i.e., gating effect; Kakigi et al., 1995; Palmer et al., 2016). However, a previous study has shown that changes in SEP to median nerve stimulation are associated with the presence of a visuo-motor conflict, but not with differences in movement kinematics (Bernier et al., 2009). Furthermore, our experimental manipulations do not impact proprioceptive cues since the distance between the two hands was kept fixed by using a mechanical frame held by participants.

Our experimental set-up allowed us to test the sense of agency for robotically-mediated movements, an aspect of bodily self-consciousness which is deeply rooted in the integration of visual and somatomotor information (Jeannerod and Pacherie, 2004; Daprati et al., 2007; Farrer et al., 2008). The decreased SoA reported during conditions with spatial and temporal conflicts as compared to conditions without any visuo-motor mismatch confirmed the classical findings (see for a review, Haggard, 2017), as well as previous results obtained using the same robotic-VR platform (Rognini et al., 2013). Although we couldn't find any relationship between the activity decrease in PPC and the reported SoA, it should be noted that parietal regions have been consistently associated with this subjective experience (Haggard, 2017). In this context, the involvement of PPC has been previously assessed in healthy individuals (Chaminade and Decety, 2002; Farrer et al., 2008; Jeannerod, 2009) and in patients with parietal lesions (Sirigu et al., 1999; Daprati et al., 2000; Ronchi et al., 2018). Converging evidence points to this area as a key player for monitoring the consistency between actions and their visual outcome (David et al., 2008). Further, the lateralization to the right PPC is compatible with previous neuroimaging evidence showing the prominent involvement of this region in the experience of controlling one's own actions, both in healthy individuals (Farrer et al., 2003) and in psychiatric and neurological patients affected by disorders of agency (Spence et al., 1997; Simeon et al., 2000). Indeed, damage to the right parietal hemisphere rather than the left one is associated not only to misattribution to one's own movements (Daprati et al., 2000), but to disturbances of other bodily self-consciousness aspects as well (Berti et al., 2005; Arzy et al., 2006; Heydrich et al., 2010).

### Limitations

The gender imbalance in our participant cohort reflects the demographics on the campus of the École Polytechnique Fédérale de Lausanne, from which we recruited our participants. Previous studies have generally not revealed any significant effect of sex on multisensory perception (basic auditory, visual and somatosensory stimuli: Hagmann and Russo, 2016; audiovisual speech: Ross et al., 2015). Thus, we have no reason to assume that our results were confounded by the gender imbalance of our cohort. The task in which participants were engaged during the experiment consisted in performing bimanual horizontal movements consecutively for 15 s, without a specific goal, that to the best of our knowledge it is unlikely to elicit gender differences at the behavioral and neurophysiological level.

Another potential limitation of the current study pertains to the relatively low number of participants. However, our sample size is the same as that of previously published studies which, like our experiment, used SEPs to investigate neural changes associated with multisensory conflicts and subjective experience (e.g., Heydrich et al., 2018; Palluel et al., 2020). Furthermore, we chose robust, data-driven approaches to data analysis, such as permutation-based statistical tests (Frossard and Renaud, 2021) and reference-independent topographical analysis of the EEG data (Murray et al., 2008). The effect sizes that we observed confirm the adequacy of our sample size (Cohen, 1988).

## Conclusion

By using a unique experimental set-up combined with electrical source imaging, we were able to uncover the neural correlates of visuo-motor integration during voluntary bimanual movement. Our results show that robotic interfaces combined with virtual reality are powerful tools for neuroscientific investigations, as they allow controlling complex dynamic interactions while introducing precise and consistent manipulations, free from potential experimenter bias. Understanding the neural substrate of robotically mediated interactions might translate into the improvement of controllability of current robotic systems and might be capitalized in neurorehabilitation applications (Mehrholz et al., 2015). Together with VR, now an established tool able to facilitate functional recovery and neural reorganization (Adamovich et al., 2010; Laver et al., 2017), EEG recordings can provide specific neural signatures to monitor changes over the course of rehabilitative interventions.

## Data availability statement

The data supporting the conclusions of this article will be made available on request by the authors, without undue reservation.

## Ethics statement

The studies involving human participants were reviewed and approved by Commission cantonale d'éthique de la recherche sur l'être humain (CER-VD). The patients/participants provided their written informed consent to participate in this study.

## Author contributions

OB conceived and supervised the research. OB, SM, and GR designed the experiment. SM carried out the experiment and wrote the manuscript. SM, FB, and MD analyzed the data. All authors contributed to the manuscript.
