# Supplementary material for: Neural signatures of visuo-motor integration during human-robot interactions
Source: Front Neurorobot. 2023 Jan 26;16:1034615. doi: 10.3389/fnbot.2022.1034615 (PMC9908758; doi:10.3389/fnbot.2022.1034615)
Supplement: Supplementary file 1 [file Data_Sheet_1.docx]

Supplementary Material

## Supplementary Figures


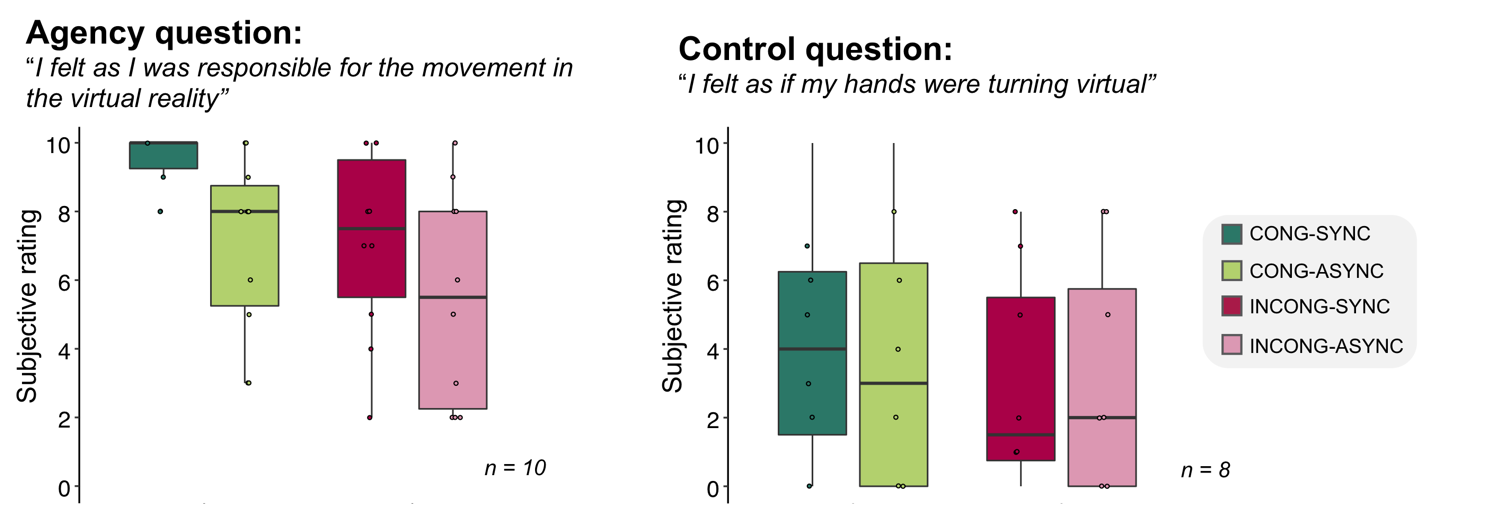


**Supplementary Figure 1.** Ratings for the questionnaire item regarding the agency question “I felt as I was responsible for the movement the virtual reality” (left) and for the control question (right) “I felt as if my hands were turning virtual” for each of the four experimental conditions. These plots have been obtained by considering data from the pool of participants whose EEG data were analyzed.
